# [*N*,*N*′-Bis(4-bromo­benzyl­idene)-2,2-di­methyl­propane-κ^2^ *N*,*N*′]iodidocopper(I)

**DOI:** 10.1107/S1600536809005078

**Published:** 2009-02-18

**Authors:** Reza Kia, Hoong-Kun Fun, Hadi Kargar

**Affiliations:** a X-ray Crystallography Unit, School of Physics, Universiti Sains Malaysia, 11800 USM, Penang, Malaysia; b Department of Chemistry, School of Science, Payame Noor University (PNU), Ardakan, Yazd, Iran

## Abstract

The title compound, [CuI(C_19_H_20_Br_2_N_2_)], lies across a crystallographic mirror plane. The coordination around the copper centre is distorted trigonal planar, with a bite angle of 94.7 (3)°. A six-membered chelate ring in a chair conformation is formed by the coordination of the imine N atoms of the bidentate ligand to the Cu^I^ atom. This conformation is required by the crystallographic mirror symmetry. The inter­planar angle between the benzene rings is 74.85 (19)°. The crystal structure exhibits weak inter­molecular C—H⋯π inter­actions, which link the mol­ecules into chains along the *b* axis.

## Related literature

For the puckering parameters, see: Cremer & Pople (1975 ▶). For related literature and the catalytic applications see, for example: Killian *et al.* (1996 ▶); Jung *et al.* (1996 ▶); Small *et al.* (1998 ▶). For a related structure, see: Kia *et al.* (2009 ▶). For the stability of the temperature controller, see Cosier & Glazer (1986 ▶).
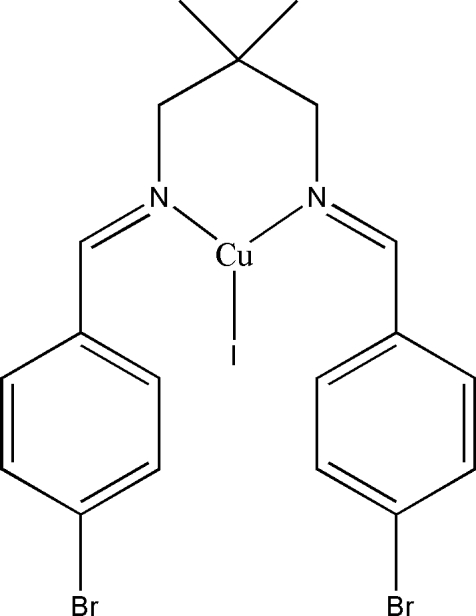


## Experimental

### 

#### Crystal data

[CuI(C_19_H_20_Br_2_N_2_)]
*M* *_r_* = 626.63
Monoclinic, 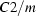

*a* = 16.2224 (15) Å
*b* = 12.2807 (12) Å
*c* = 10.6292 (12) Å
β = 91.599 (6)°
*V* = 2116.8 (4) Å^3^
*Z* = 4
Mo *K*α radiation
μ = 6.27 mm^−1^
*T* = 100 K
0.58 × 0.09 × 0.05 mm

#### Data collection

Bruker SMART APEXII CCD area-detector diffractometer
Absorption correction: multi-scan (**SADABS**; Bruker, 2005 ▶) *T*_min_ = 0.119, *T*_max_ = 0.714
10374 measured reflections
1936 independent reflections
1474 reflections with *I* > 2σ(*I*)
*R*_int_ = 0.099

#### Refinement

*R*[*F* ^2^ > 2σ(*F* ^2^)] = 0.050
*wR*(*F* ^2^) = 0.112
*S* = 1.16
1936 reflections
121 parameters
H-atom parameters constrained
Δρ_max_ = 2.64 e Å^−3^
Δρ_min_ = −0.99 e Å^−3^

### 

Data collection: *APEX2* (Bruker, 2005 ▶); cell refinement: *APEX2*; data reduction: *SAINT* (Bruker, 2005 ▶); program(s) used to solve structure: *SHELXTL* (Sheldrick, 2008 ▶); program(s) used to refine structure: *SHELXTL*; molecular graphics: *SHELXTL*; software used to prepare material for publication: *SHELXTL* and *PLATON* (Spek, 2009 ▶).

## Supplementary Material

Crystal structure: contains datablocks global, I. DOI: 10.1107/S1600536809005078/ez2161sup1.cif

Structure factors: contains datablocks I. DOI: 10.1107/S1600536809005078/ez2161Isup2.hkl

Additional supplementary materials:  crystallographic information; 3D view; checkCIF report

## Figures and Tables

**Table 1:** Hydrogen-bond geometry (Å, °) *Cg*1 is the centroid of the C1–C6 benzene ring.

*D*—H⋯*A*	*D*—H	H⋯*A*	*D*⋯*A*	*D*—H⋯*A*
C8—H8*A*⋯*Cg*1^i^	0.99	2.83	3.631 (9)	138

Symmetry code: (i) 
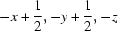
.

**Fig. 1.**
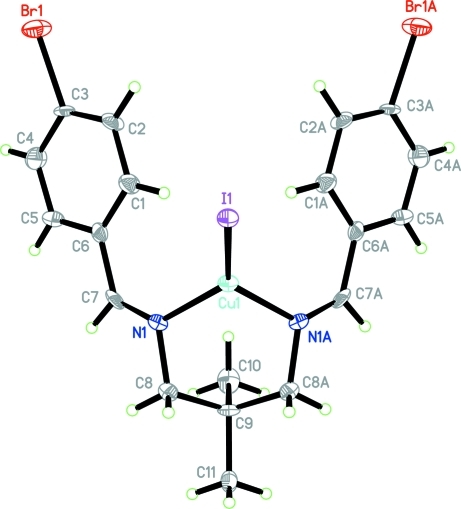
The molecular structure of (I), showing 40% probability displacement ellipsoids and the atomic numbering. Symmetry code for A atoms; x, -y + 1, z.

**Fig. 2.**
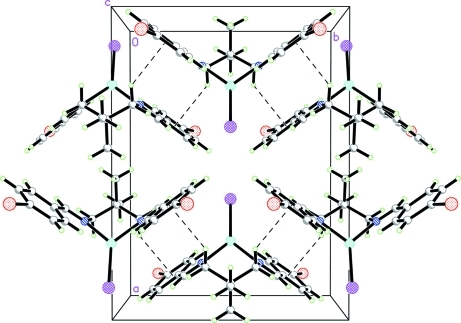
The crystal packing of (I), viewed down the c-axis, showing C—H···π interactions linking the molecules into chains along the b-axis.

## supplementary crystallographic information

### Comment

In recent years, an increasing amount of research has been focused on the design and preparation of mono- or dinuclear mixed ligand transition metal complexes with neutral, chelating nitrogen-containing ligands. Early and late transition metal complexes of this type have been extensively used as catalysts for a wide categories of reactions, including olefin polymerization (Killian *et al.*, 1996) and oxygen activation (Jung *et al.*, 1996). In this context, diverse chelating Schiff base type ligands, amines and pyridine derivatives (Small *et al.*, 1998) have successfully been applied in the preparation of these homogeneous catalysts. Here we report the crystal structure of an aldimine Schiff base ligand with copper(I) iodide. To the best of our knowledge, only one such related compound has been published (Kia *et al.*, 2009). The title compound is the second tricoordinate complex of an aldimine bis-Schiff base ligand with copper(I) iodide adopting trigonal planar geometry.

The title compound, I, Fig. 1, lies across a crystallographic mirror plane. Atoms I1, Cu1, C9, C10 and C11 lies on this mirror plane. The asymmetric unit of (I) is composed of one-half of the molecule. The coordination around the copper centre is distorted trigonal planar, with a bite angle of 94.7 (3)°. The deviation of the Cu atom from the N1/N1A/I1 plane is -0.105 (4) Å. A six-membered chelate ring with a chair conformation is formed by the coordination of iminic N atoms of the bidentate ligand to the Cu(I) atom, with ring puckering parameters (Cremer & Pople, 1975) of Q = 0.696 (7) Å, Θ = 172.2 (6)°, Φ = 180 (5)°. This conformation is required if the local symmetry of the metal coordination site is in accordance with the mirror plane that passes through the metal atom normal to the line connecting the nitrogen atoms. The dihedral angle between the phenyl rings is 74.85 (19)°. The crystal structure is stabilized by weak intermolecular C—H···π interactions (*Cg*1 is the centroid of the C1–C6 benzene ring) which link the molecules into chains along the *b*-axis (Fig. 2 and Table 1).

### Experimental

*N*,*N*'-Bis(4-bromobenzylidene)-2,2-dimethylpropane (783 mg, 2 mmol) was added dropwise to a suspension of CuI (380 mg, 2.0 mmol) in 50 ml of THF. After 15 min a clear yellowish solution was obtained. The volume of the reaction mixture was reduced until the formation of a yellow precipitate occurred. Single crystals suitable for X-ray diffraction were grown from the acetonitrile solution.

### Refinement

All H atoms were positioned geometrically with C—H = 0.95–0.99 Å and refined in a riding model approximation with *U*_iso_(H) = 1.2*U*_eq_(C). The highest peak (2.64 e. Å^-3^) is located 1.02 Å from I1 and the deepest hole (-0.99 e. Å^-3^) is located 0.58 Å from H10A.

### Crystal data

**Table:**

[CuI(C_19_H_20_Br_2_N_2_)]	*F*(000) = 1200
*M**_r_* = 626.63	*D*_x_ = 1.966 Mg m^−^^3^
Monoclinic, *C*2/*m*	Mo *K*α radiation, λ = 0.71073 Å
Hall symbol: -C 2y	Cell parameters from 4080 reflections
*a* = 16.2224 (15) Å	θ = 2.5–29.5°
*b* = 12.2807 (12) Å	µ = 6.27 mm^−^^1^
*c* = 10.6292 (12) Å	*T* = 100 K
β = 91.599 (6)°	Needle, yellow
*V* = 2116.8 (4) Å^3^	0.58 × 0.09 × 0.05 mm
*Z* = 4	

### Data collection

**Table:**

Bruker SMART APEXII CCD area-detector diffractometer	1936 independent reflections
Radiation source: fine-focus sealed tube	1474 reflections with *I* > 2σ(*I*)
graphite	*R*_int_ = 0.099
φ and ω scans	θ_max_ = 25.0°, θ_min_ = 2.1°
Absorption correction: multi-scan (*SADABS*; Bruker, 2005)	*h* = −19→19
*T*_min_ = 0.119, *T*_max_ = 0.714	*k* = −14→14
10374 measured reflections	*l* = −12→12

### Refinement

**Table:**

Refinement on *F*^2^	Primary atom site location: structure-invariant direct methods
Least-squares matrix: full	Secondary atom site location: difference Fourier map
*R*[*F*^2^ > 2σ(*F*^2^)] = 0.050	Hydrogen site location: inferred from neighbouring sites
*wR*(*F*^2^) = 0.112	H-atom parameters constrained
*S* = 1.16	*w* = 1/[σ^2^(*F*_o_^2^) + (0.0405*P*)^2^ + 16.0151*P*] where *P* = (*F*_o_^2^ + 2*F*_c_^2^)/3
1936 reflections	(Δ/σ)_max_ < 0.001
121 parameters	Δρ_max_ = 2.64 e Å^−^^3^
0 restraints	Δρ_min_ = −0.99 e Å^−^^3^

### Special details

**Table:**

Experimental. The crystal was placed in the cold stream of an Oxford Cryosystems Cobra open-flow nitrogen cryostat (Cosier & Glazer, 1986) operating at 100.0 (1) K.
Geometry. All esds (except the esd in the dihedral angle between two l.s. planes) are estimated using the full covariance matrix. The cell esds are taken into account individually in the estimation of esds in distances, angles and torsion angles; correlations between esds in cell parameters are only used when they are defined by crystal symmetry. An approximate (isotropic) treatment of cell esds is used for estimating esds involving l.s. planes.
Refinement. Refinement of F^2^ against ALL reflections. The weighted R-factor wR and goodness of fit S are based on F^2^, conventional R-factors R are based on F, with F set to zero for negative F^2^. The threshold expression of F^2^ > 2sigma(F^2^) is used only for calculating R-factors(gt) etc. and is not relevant to the choice of reflections for refinement. R-factors based on F^2^ are statistically about twice as large as those based on F, and R- factors based on ALL data will be even larger.

### Fractional atomic coordinates and isotropic or equivalent isotropic displacement parameters (Å^2^)

**Table:**

	*x*	*y*	*z*	*U*_iso_*/*U*_eq_	
I1	0.38486 (4)	0.5000	0.14862 (6)	0.0166 (2)	
Br1	0.11472 (5)	0.16378 (8)	0.54336 (7)	0.0309 (3)	
Cu1	0.25251 (8)	0.5000	0.02730 (11)	0.0168 (3)	
N1	0.1853 (3)	0.3798 (6)	−0.0519 (5)	0.0167 (14)	
C1	0.1751 (5)	0.3315 (7)	0.2223 (7)	0.0213 (18)	
H1A	0.2085	0.3923	0.2022	0.026*	
C2	0.1683 (4)	0.3023 (7)	0.3463 (7)	0.0210 (18)	
H2A	0.1957	0.3426	0.4113	0.025*	
C3	0.1204 (5)	0.2130 (7)	0.3742 (6)	0.0193 (18)	
C4	0.0779 (5)	0.1560 (7)	0.2811 (8)	0.026 (2)	
H4A	0.0442	0.0958	0.3021	0.031*	
C5	0.0852 (4)	0.1878 (7)	0.1556 (7)	0.0188 (18)	
H5A	0.0559	0.1496	0.0907	0.023*	
C6	0.1354 (4)	0.2756 (7)	0.1260 (7)	0.0172 (18)	
C7	0.1428 (4)	0.3030 (7)	−0.0085 (7)	0.0184 (17)	
H7A	0.1130	0.2590	−0.0675	0.022*	
C8	0.1816 (5)	0.3967 (7)	−0.1892 (7)	0.0213 (18)	
H8A	0.1542	0.3330	−0.2292	0.026*	
H8B	0.2387	0.3997	−0.2198	0.026*	
C9	0.1360 (7)	0.5000	−0.2327 (9)	0.020 (3)	
C10	0.0477 (6)	0.5000	−0.1869 (11)	0.028 (3)	
H10A	0.0492	0.5000	−0.0947	0.041*	
H10B	0.0187	0.4348	−0.2177	0.041*	
C11	0.1346 (7)	0.5000	−0.3771 (10)	0.024 (3)	
H11A	0.1916	0.5000	−0.4055	0.036*	
H11B	0.1061	0.5652	−0.4091	0.036*	

### Atomic displacement parameters (Å^2^)

**Table:**

	*U*^11^	*U*^22^	*U*^33^	*U*^12^	*U*^13^	*U*^23^
I1	0.0191 (4)	0.0171 (4)	0.0134 (4)	0.000	−0.0029 (3)	0.000
Br1	0.0371 (5)	0.0400 (6)	0.0154 (4)	−0.0155 (4)	−0.0026 (3)	0.0048 (4)
Cu1	0.0195 (7)	0.0188 (8)	0.0121 (7)	0.000	−0.0020 (5)	0.000
N1	0.020 (3)	0.018 (4)	0.012 (3)	0.001 (3)	0.001 (2)	−0.002 (3)
C1	0.024 (4)	0.021 (5)	0.019 (4)	−0.007 (3)	0.002 (3)	−0.003 (4)
C2	0.025 (4)	0.023 (5)	0.015 (4)	−0.009 (4)	−0.004 (3)	−0.007 (4)
C3	0.031 (4)	0.022 (5)	0.005 (4)	−0.003 (4)	0.001 (3)	0.002 (3)
C4	0.031 (5)	0.022 (5)	0.025 (5)	−0.010 (4)	0.002 (4)	0.003 (4)
C5	0.018 (4)	0.025 (5)	0.013 (4)	−0.001 (3)	−0.002 (3)	−0.002 (4)
C6	0.019 (4)	0.014 (5)	0.020 (4)	0.005 (3)	0.004 (3)	−0.002 (4)
C7	0.022 (4)	0.016 (5)	0.017 (4)	0.000 (3)	−0.005 (3)	−0.010 (4)
C8	0.031 (4)	0.019 (5)	0.014 (4)	−0.003 (4)	0.001 (3)	−0.002 (4)
C9	0.029 (6)	0.024 (7)	0.007 (5)	0.000	0.003 (4)	0.000
C10	0.022 (6)	0.034 (8)	0.027 (7)	0.000	−0.009 (5)	0.000
C11	0.038 (7)	0.020 (7)	0.015 (6)	0.000	−0.006 (5)	0.000

### Geometric parameters (Å, °)

**Table:**

I1—Cu1	2.4735 (14)	C5—C6	1.392 (11)
Br1—C3	1.902 (7)	C5—H5A	0.9500
Cu1—N1	2.006 (6)	C6—C7	1.477 (11)
Cu1—N1^i^	2.006 (6)	C7—H7A	0.9500
N1—C7	1.263 (10)	C8—C9	1.533 (10)
N1—C8	1.474 (9)	C8—H8A	0.9900
C1—C2	1.374 (11)	C8—H8B	0.9900
C1—C6	1.378 (10)	C9—C10	1.527 (15)
C1—H1A	0.9500	C9—C8^i^	1.533 (10)
C2—C3	1.381 (11)	C9—C11	1.535 (14)
C2—H2A	0.9500	C10—H10A	0.9800
C3—C4	1.381 (11)	C10—H10B	0.9800
C4—C5	1.398 (11)	C11—H11A	0.9800
C4—H4A	0.9500	C11—H11B	0.9801
			
N1—Cu1—N1^i^	94.8 (4)	C5—C6—C7	117.3 (7)
N1—Cu1—I1	132.24 (18)	N1—C7—C6	125.7 (7)
N1^i^—Cu1—I1	132.24 (18)	N1—C7—H7A	117.1
C7—N1—C8	117.4 (6)	C6—C7—H7A	117.1
C7—N1—Cu1	133.8 (5)	N1—C8—C9	114.9 (7)
C8—N1—Cu1	108.5 (5)	N1—C8—H8A	108.5
C2—C1—C6	122.3 (8)	C9—C8—H8A	108.5
C2—C1—H1A	118.9	N1—C8—H8B	108.5
C6—C1—H1A	118.9	C9—C8—H8B	108.5
C1—C2—C3	118.3 (7)	H8A—C8—H8B	107.5
C1—C2—H2A	120.9	C10—C9—C8^i^	110.7 (6)
C3—C2—H2A	120.9	C10—C9—C8	110.7 (6)
C4—C3—C2	121.4 (7)	C8^i^—C9—C8	111.7 (9)
C4—C3—Br1	118.7 (6)	C10—C9—C11	109.3 (9)
C2—C3—Br1	119.8 (6)	C8^i^—C9—C11	107.1 (6)
C3—C4—C5	119.2 (8)	C8—C9—C11	107.1 (6)
C3—C4—H4A	120.4	C9—C10—H10A	108.7
C5—C4—H4A	120.4	C9—C10—H10B	109.8
C6—C5—C4	119.8 (7)	H10A—C10—H10B	109.5
C6—C5—H5A	120.1	C9—C11—H11A	108.6
C4—C5—H5A	120.1	C9—C11—H11B	109.9
C1—C6—C5	118.9 (7)	H11A—C11—H11B	109.5
C1—C6—C7	123.8 (7)		
			
N1^i^—Cu1—N1—C7	−119.0 (7)	C4—C5—C6—C1	−1.9 (11)
I1—Cu1—N1—C7	70.2 (8)	C4—C5—C6—C7	178.0 (7)
N1^i^—Cu1—N1—C8	54.2 (5)	C8—N1—C7—C6	−177.4 (7)
I1—Cu1—N1—C8	−116.5 (4)	Cu1—N1—C7—C6	−4.5 (12)
C6—C1—C2—C3	0.8 (12)	C1—C6—C7—N1	−0.3 (12)
C1—C2—C3—C4	−2.2 (12)	C5—C6—C7—N1	179.8 (7)
C1—C2—C3—Br1	175.9 (6)	C7—N1—C8—C9	109.6 (8)
C2—C3—C4—C5	1.6 (13)	Cu1—N1—C8—C9	−64.9 (7)
Br1—C3—C4—C5	−176.6 (6)	N1—C8—C9—C10	−57.8 (9)
C3—C4—C5—C6	0.5 (12)	N1—C8—C9—C8^i^	66.0 (10)
C2—C1—C6—C5	1.3 (12)	N1—C8—C9—C11	−176.9 (7)
C2—C1—C6—C7	−178.6 (7)		

Symmetry codes: (i) *x*, −*y*+1, *z*.

### Hydrogen-bond geometry (Å, °)

**Table:**

*D*—H···*A*	*D*—H	H···*A*	*D*···*A*	*D*—H···*A*
C8—H8A···Cg1^ii^	0.99	2.83	3.631 (9)	138

Symmetry codes: (ii) −*x*+1/2, −*y*+1/2, −*z*.
