# [*N*,*N*′-Bis(4-bromo­benzyl­idene)-2,2-di­methyl­propane-κ^2^ *N*,*N*′]iodidocopper(I). Corrigendum

**DOI:** 10.1107/S1600536809023307

**Published:** 2009-06-27

**Authors:** Reza Kia, Hoong-Kun Fun, Hadi Kargar

**Affiliations:** a X-ray Crystallography Unit, School of Physics, Universiti Sains Malaysia, 11800 USM, Penang, Malaysia; b Department of Chemistry, School of Science, Payame Noor University (PNU), Ardakan, Yazd, Iran

## Abstract

The chemical name of the title compound in the paper by Kia, Fun & Kargar [*Acta Cryst.* (2009), E**65**, m289] is corrected.

## Abstract 2

Corrigendum to *Acta Cryst.* (2009), E**65**, m289.

In the paper by Kia, Fun & Kargar [*Acta Cryst.* (2009), E**65**, m289], the chemical name given in the *Title* should be ‘[*N*,*N*′-Bis(4-bromo­benzyl­idene)-2,2-di­methyl­propane-1,3-diamine-κ^2^ *N*,*N*′]iodidocopper(I)’.

